# Morocco as a possible source for acquisition of *Rhinocladiella mackenziei*

**DOI:** 10.1371/journal.pntd.0009563

**Published:** 2021-08-19

**Authors:** Baptiste Lafont Rapnouil, Jérémie F. Cohen, Eric Bailly, Louis Bernard, Dea Garcia-Hermoso, Fanny Lanternier, Catherine Horodyckid, Lucie Limousin, Ephrem Salamé, Ilyess Zemmoura, Guillaume Desoubeaux, Olivier Lortholary

**Affiliations:** 1 Université de Paris, Necker-Pasteur Center for Infectious Diseases and Tropical Medicine, Necker-Enfants Malades Hospital, APHP, Imagine Institute, Paris, France; 2 Inserm U1153, Centre of Research in Epidemiology and Statistics (CRESS), Université de Paris, Paris, France; 3 Parasitology, Mycology, and Tropical Diseases Department, Bretonneau Hospital, Tours, France; 4 Department of Infectious Diseases, Bretonneau Hospital, Tours, France; 5 Institut Pasteur, Molecular Mycology Unit, National Reference Center for Invasive Mycoses and Antifungals (NRCMA), UMR 2000, CNRS, Paris, France; 6 Department of Neurosurgery, Foch Hospital, Suresnes, France; 7 Department of Microbiology, Foch Hospital, Suresnes, France; 8 Trousseau Hospital, Chambray-lès-Tours, France; 9 Department of Neurosurgery, CHRU de Tours, Tours, France; 10 Université de Tours, CEPR—INSERM U1100/Équipe 3, Faculté de Médecine, Tours, France; National Institute for Communicable Diseases, Johannesburg, South Africa, SOUTH AFRICA

## Abstract

*Rhinocladiella mackenziei* cerebral phaeohyphomycosis is a rare severe disease that has been typically described in the Middle East. Here, we report 2 cases of *R*. *mackenziei* cerebral phaeohyphomycosis in patients from Morocco, diagnosed and treated in France, and raise a concern about the ever-going extension of the area at risk for this devastating invasive fungal infection.

## Introduction

Cerebral phaeohyphomycosis is a rare but severe infection due to melanized fungi. Along with *Cladophialophora bantiana*, *Rhinocladiella mackenziei* is one of the main fungi responsible for this disease [1]. There are no definite risk factors for this pathogen, and, notably, no immunodeficiency found in most of the afflicted patients [2]. On 2% malt agar, *R*. *mackenziei* grows as green olive to brown lanose colonies. The microscopic aspect is characterized by the presence of short conidiophores arising from melanized septate hyphae. In addition, hyaline denticled–conidiogenous cells producing pale brown ellipsoidal conidia in sympodial succession are observed. Prognosis of cerebral phaeohyphomycosis is dreadful, with only a handful of nonlethal cases reported [3–6]. Other members of the *Rhinocladiella* genus are involved in human diseases, such as chromoblastomycosis [7]. Diagnosis has become more accessible and accurate with the advent of polymerase chain reaction (PCR) techniques and DNA sequencing, which allowed to pin down the precise taxonomy of black fungi [8].

While other pathogens involved in cerebral phaeohyphomycosis seem to be found worldwide [1], *R*. *mackenziei* has classically been reported as being geographically restricted to the Middle East. However, in the last decade, cases were reported in a broader area [4,9,10]. The aim of this report is to underline the extension of the endemic areas for *R*. *mackenziei* by reporting the first 2 cases of *R*. *mackenziei* cerebral phaeohyphomycosis in patients from Morocco.

## Case presentation

### Case 1

A 60-year-old woman from Morocco, living in France, with a past medical history of tuberculosis and cirrhosis secondary to hepatitis B and C infections, underwent liver transplantation in February 2014 because of refractory ascites and recurrent encephalopathy. Postoperative recovery was uneventful except for episodes of tremor, explored by a cerebral computerized tomography (CT) scan in March 2014, which was normal. In May, she presented a generalized tonic–clonic seizure, followed by a left upper limb paresis. The cerebral CT scan showed a 16-mm right frontal ring–enhancing lesion, and the magnetic resonance imaging (MRI) confirmed the suspicion of a cerebral abscess (**S1A Fig**). A stereotactic guided biopsy was performed. Histopathology found pigmented fungal hyphae (**S1B Fig**), and the patient was started on flucytosine (80 mg/kg in 4 divided doses)–liposomal amphotericin B (AmB-L, 3.3 mg/kg in 1-hour infusion) combination therapy. AmB-L was then switched to voriconazole (4 mg/kg intravenously twice a day) because of renal toxicity. The clinical course was favorable, with partial recovery of neurological deficits. The French National Reference Center for Invasive Mycoses and Antifungals (NRCMA; Institut Pasteur, Paris) identified *R*. *mackenziei* based on morphological features under Biosafety *Level 3* (BSL-3) regulations and sequencing of the ITS1-5.8S-ITS2 region of rDNA (GenBank accession number: MZ128136). DNA extraction method [11] and PCR protocol [12] have been described elsewhere. MRI reevaluation in August 2014 showed expansion of the lesion and related edema. Posaconazole (2.7 mg/kg oral suspension 4 times a day) was added to the treatment regimen, and immunosuppression was lowered. She deteriorated quickly with repeated seizures, right hemiplegia, and intracranial hypertension. Voriconazole was switched back to AmB-L, and rescue surgery was attempted to no avail. The patient expired 6 months after the diagnosis.

### Case 2

As briefly reported elsewhere [13], this patient was a 59-year-old woman from Morocco who had been living in France for 30 years. She had been to Morocco for the last time in October 2017 and had no other travel history. Her past medical history was notable for ulcerative ileocolitis treated by prednisone (30 mg/day) and azathioprine since October 2017 because of presumed Crohn disease and suspicion of pulmonary tuberculosis. She presented in July 2018 with a 2-week history of memory loss, left-sided headaches, dysphasia, mild photo-phonophobia, vertigo, and right arm neuropathic pain, without motor deficit. A cerebral CT scan revealed a 36-mm–long axis temporoparietal lesion compatible with a brain abscess, with significant perilesional edema. She underwent stereotactic guided biopsy of the lesion. Direct examination showed black fungal hyphae. She was started on cefotaxime, metronidazole, and voriconazole (4 mg/kg intravenously twice daily), and azathioprine was stopped. Seven-day CT reevaluation showed no lesion reduction. AmB-L (5.7 mg/kg once a day intravenously) and flucytosine (30 mg/kg 4 times a day intravenously) were added to voriconazole. The isolate sent to the NRCMA for identification revealed *R*. *mackenziei* after morphological examination under BSL-*3* regulations and sequencing of the ITS1-5.8S-ITS2 region of rDNA (GenBank accession number: MZ128137). Antifungal therapy was switched to posaconazole only (5.7 mg/kg once a day orally). MRI confirmed the temporoparietal abscess and showed associated lateral sinus venous thrombosis. She underwent surgery with incomplete resection of the lesion due to left lateral sinus invasion. She experienced regression of dysphasia and headaches and recovery of better memory function. The cause of ileocolitis was revised as being *Mycobacterium tuberculosis*, with PCR confirmation, and the patient was treated for 12 months using a moxifloxacin-based antimycobacterial regimen. Corticosteroids were progressively tapered and stopped. After 26 months of follow-up, the patient is clinically stable, and MRI reevaluations showed no relapse of cerebral phaeohyphomycosis. In addition to drug-induced immunosuppression (i.e., steroids and azathioprine), that patient had no known genetic predisposition for invasive fungal disease (based on sequencing of IL17F, IL17RA, IL17RC, ACT1, JNK1, STAT1, STAT3, RORC, ZNF341, CARD9, and AIRE genes).

## Case discussion

Phaeohyphomycosis is a neglected fungal disease. *R*. *mackenziei* is one of the responsible pathogens, with the particularity of being considered geographically restricted. Here, we report, to our knowledge, the first 2 cases from Morocco natives.

Risk factors for *R*. *mackenziei* infection remain unclear. Although some cases occurred in immunocompromised hosts such as solid organ transplant recipients or patients with hematological malignancies, other cases were reported in patients with few comorbidities [1]. Little is known about the reservoir of *R*. *mackenziei* and the route of contamination, and it was never isolated from the environment [14].

**Fig 1:**
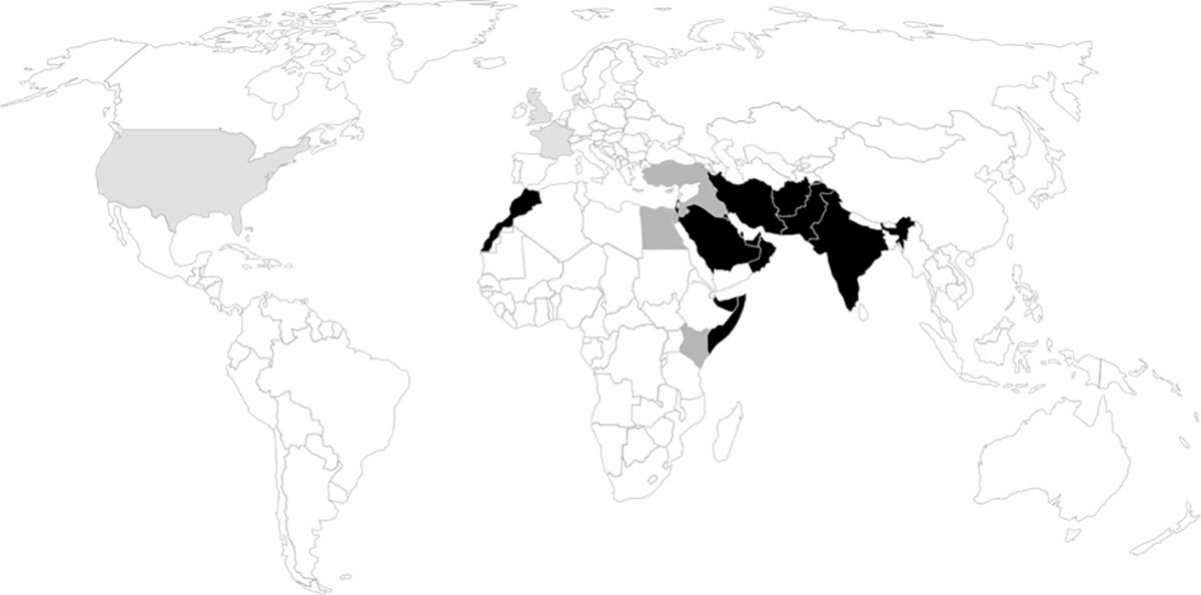
Geographical distribution of *Rhinocladiella mackenziei* cerebral phaeohyphomycosis (*N* = 36). In black, countries with confirmed cases. In dark gray, countries with possible contamination. In light gray, countries where diagnoses were made but unlikely to be at risk for contamination (probable imported cases). If several countries were possibly incriminated, priority was given to the country with other confirmed cases if present; else, every country was considered as “possible contamination.” Articles used in this figure are reported in **S1 Table**. Map template can be found at https://www.freeworldmaps.net/powerpoint/.

So far, most of the cases found in the literature originated from the Middle East (24/36, 67%; **Fig 1**, **S1 Table**). However, recently, an increasing number of cases have been reported from other countries at risk, notably Pakistan, India, Iran [15], and, now, Morocco, which still concurs with the hypothesis of an organism fitted for an arid climate. Similar organisms have been isolated from air biofilters, dry, and/or acidic soil [16]. An analysis of the literature allows formulating new hypotheses about the pathogenesis of *R*. *mackenziei* cerebral phaeohyphomycosis. Reading the case from Cristini and colleagues [10], with a 20-year gap between the patient’s stay in Afghanistan and the disease, one can assume the possibility of a latent in vivo reservoir, which ultimately reactivates under particular circumstances. Many patients in the literature have a travel history to areas with arid climates. If we hypothesize a prolonged carrier state, the geographical area at risk could be even broader than what previously thought: Turkey, Kenya, Egypt, and Somalia might also be at risk, for example (**Fig 1**).

Shortly, we can recommend extensive surgery as the cornerstone for treatment. Posaconazole is probably the optimal associated antifungal for *R*. *mackenziei*, based on our one surviving patient and success of others with this triazole [3,10]. These conclusions are limited by the scarce available clinical evidence, but concordant with in vitro [17] and animal data [18].

This study has limitations. First, this was not an exhaustive collection of all imported French cases of *R*. *mackenziei* infections, exposing to selection bias. Moreover, the Necker-Enfants Malades Hospital is associated with the NRCMA and may have a different case mix than other centers. Second, this is a retrospective study, limiting the conclusions that can be made about exposure to the pathogen and route of contamination. Third, the small number of cases obviously limits the generalizability of our findings.

In conclusion, the area at risk of *R*. *mackenziei* cerebral abscess is extending to new countries and now also includes North Africa with Morocco. Clinicians should be aware of this emerging disease and take a detailed travel history in patients with atypical and undocumented brain abscess.

### Ethics

Informed consent could not be obtained for the patient of case 1 (deceased). In conformity with data protection regulations, noncritical information was modified to ensure pseudonymization. The Assistance publique Hôpitaux de Paris Ethical Research Committee (CERAPHP Centre, Institutional Review Board [IRB] registration number #00011928) reviewed the manuscript and provided a waiver for consent (Ref 2020-11-17). Formal written informed consent was obtained from the patient of case 2 to use her medical information and specimens for medical and scientific purposes aiming at improving medical knowledge and patient care.

**Key Learning Points**

- *Rhinocladiella mackenziei* cerebral phaeohypohmycosis is a rare and severe infection.
- Immunocompetent hosts are at risk.
- The geographic area at risk for this infection is growing.
- Effective treatment probably requires surgery associated with antifungal therapy.

## Supporting information

S1 Fig Diagnostic material of case 1. **(A)** Contrast-enhanced T1 gadolinium MRI showing a ring-enhancing lesion compatible with an abscess in the right frontal lobe. **(B)** HES stain of an aspirate of the brain lesion shown in (A), magnified 500×. Note the microscopic features that can suggest phaeohyphomycosis: branched (gray dart) pigmented filaments with visible septate (black arrow). The specific morphological features of *Rhinocladiella mackenziei* have been well described elsewhere [2,8,9]. HES, hematoxylin–eosin saffron; MRI, magnetic resonance imaging.
(TIF)

S1 Table Cases reported in Fig 1. To identify cases, we searched in PubMed using the following keywords: “*Rhinocladiella mackenzieie*,” “*Ramichloridium obovoideum*,” “*Ramichloridium mackenziei*,” and “*Ramichloridium obovoidea*.” We searched from database inception onward without language restrictions. The last search was conducted on May 15, 2020.
(DOCX)

## References

[1] Revankar SG, Sutton DA, Rinaldi MG. Primary Central Nervous System Phaeohyphomycosis: A Review of 101 Cases. Clin Infect Dis. 2004;38:206–16. doi: 10.1086/380635 14699452

[2] Revankar SG, Sutton DA. Melanized Fungi in Human Disease. Clin Microbiol Rev. 2010;23:884–928. doi: 10.1128/CMR.00019-10 20930077 PMC2952981

[3] Al-abdely HM, Alkhunaizi AM, Al-tawfiq JA, Hassounah M, Rinaldi MG, Sutton DA. Successful therapy of cerebral phaeohyphomycosis due to Ramichloridium mackenziei with the new triazole posaconazole. Med Mycol. 2005;43:91–5. doi: 10.1080/13693780400011104 15712614

[4] Jabeen K, Farooqi J, Zafar A, Jamil B, Faisal Mahmood S, Ali F, et al. Rhinocladiella mackenziei as an Emerging Cause of Cerebral Phaeohyphomycosis in Pakistan: A Case Series. Clin Infect Dis. 2011;52:213–7. doi: 10.1093/cid/ciq114 21288846

[5] Yusupov N, Merve A, Warrell CE, Johnson E, Curtis C, Samandouras G. Multiple brain abscesses caused by Rhinocladiella mackenziei in an immunocompetent patient: a case report and literature review. Acta Neurochir. 2017;159:1757–63. doi: 10.1007/s00701-017-3141-0 28365816

[6] Hardman N, Young N, Hobson R, Sandoe J, Wellberry-Smith M, Thomson S, et al. Prolonged survival after disseminated *Rhinocladiella* infection treated with surgical excision and posaconazole. Transpl Infect Dis. 2020;22. doi: 10.1111/tid.13264 32053285

[7] Badali H, Bonifaz A, Barrón-Tapia T, Vázquez-González D, Estrada-Aguilar L, Cavalcante Oliveira NM, et al. Rhinocladiella aquaspersa, proven agent of verrucous skin infection and a novel type of chromoblastomycosis. Med Mycol. 2010;48:696–703. doi: 10.3109/13693780903471073 20055741

[8] Arzanlou M, Groenewald JZ, Gams W, Braun U, Shin H-D, Crous PW. Phylogenetic and morphotaxonomic revision of Ramichloridium and allied genera. Stud Mycol. 2007;58:57–93. doi: 10.3114/sim.2007.58.03 18490996 PMC2104745

[9] Badali H, Chander J, Bansal S, Aher A, Borkar SS, Meis JF, et al. First Autochthonous Case of Rhinocladiella mackenziei Cerebral Abscess Outside the Middle East. J Clin Microbiol. 2010;48:646–9. doi: 10.1128/JCM.01855-09 20007402 PMC2815588

[10] Cristini A, Garcia-Hermoso D, Celard M, Albrand G, Lortholary O. Cerebral Phaeohyphomycosis Caused by Rhinocladiella mackenziei in a Woman Native to Afghanistan. J Clin Microbiol. 2010;48:3451–4. doi: 10.1128/JCM.00924-10 20592148 PMC2937739

[11] Garcia-Hermoso D, Hamane S, Fekkar A, Jabet A, Denis B, Siguier M, et al. Invasive Infections with Nannizziopsis obscura Species Complex in 9 Patients from West Africa, France, 2004–2020. Emerg Infect Dis. 2020;26. doi: 10.3201/eid2609.200276 32819454 PMC7454062

[12] Garcia-Hermoso D, Hoinard D, Gantier J-C, Grenouillet F, Dromer F, Dannaoui E. Molecular and phenotypic evaluation of Lichtheimia corymbifera (formerly Absidia corymbifera) complex isolates associated with human mucormycosis: rehabilitation of *L*. *ramosa*. J Clin Microbiol. 2009;47:3862–70. doi: 10.1128/JCM.02094-08 19759217 PMC2786664

[13] Barde F, Billaud E, Goldwirt L, Horodyckid C, Jullien V, Lanternier F, et al. Low Central Nervous System Posaconazole Concentrations during Cerebral Phaeohyphomycosis. Antimicrob Agents Chemother. 2019;63:e01184–19. doi: 10.1128/AAC.01184-19 31427294 PMC6811437

[14] Moreno LF, Ahmed AAO, Brankovics B, Cuomo CA, Menken SBJ, Taj-Aldeen SJ, et al. Genomic Understanding of an Infectious Brain Disease from the Desert. G3 (Bethesda). 2018;8:909–22. doi: 10.1534/g3.117.300421 29326229 PMC5844311

[15] Mohammadi R, Mohammadi A, Ashtari F, Khorvash F, Hakamifard A, Vaezi A, et al. Cerebral phaeohyphomycosis due to *Rhinocladiella mackenziei* in Persian Gulf region: A case and review. Mycoses. 2018;61:261–5. doi: 10.1111/myc.12734 29205524

[16] Badali H, Prenafeta-Boldu FX, Guarro J, Klaassen CH, Meis JF, de Hoog GS. Cladophialophora psammophila, a novel species of Chaetothyriales with a potential use in the bioremediation of volatile aromatic hydrocarbons. Fungal Biol. 2011;115:1019–29. doi: 10.1016/j.funbio.2011.04.005 21944214

[17] Badali H, de Hoog GS, Curfs-Breuker I, Meis JF. In vitro activities of antifungal drugs against Rhinocladiella mackenziei, an agent of fatal brain infection. J Antimicrob Chemother. 2010;65:175–7. doi: 10.1093/jac/dkp390 19854862

[18] Al-Abdely HM, Najvar L, Bocanegra R, Fothergill A, Loebenberg D, Rinaldi MG, et al. SCH 56592, Amphotericin B, or Itraconazole Therapy of Experimental Murine Cerebral Phaeohyphomycosis Due to Ramichloridium obovoideum (“Ramichloridium mackenziei”). Antimicrob Agents Chemother. 2000;44:1159–62. doi: 10.1128/AAC.44.5.1159-1162.2000 10770745 PMC89838

