# Supplementary figures and images for: Morocco as a possible source for acquisition of Rhinocladiella mackenziei
Source: PLoS Negl Trop Dis. 2021 Aug 19;15(8):e0009563. doi: 10.1371/journal.pntd.0009563 (PMC8376069; doi:10.1371/journal.pntd.0009563)

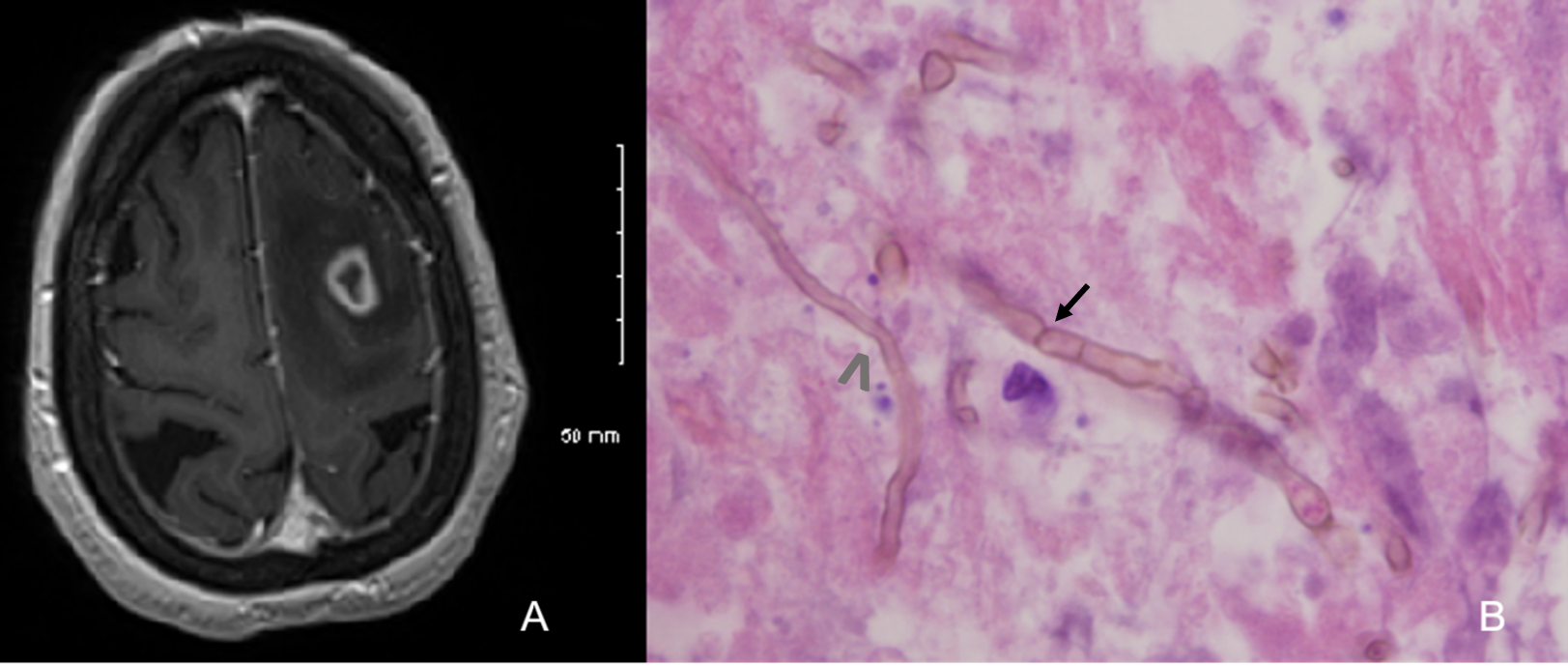

Supplement: S1 Fig — (A) Contrast-enhanced T1 gadolinium MRI showing a ring-enhancing lesion compatible with an abscess in the right frontal lobe. (B) HES stain of an aspirate of the brain lesion shown in (A), magnified 500×. Note the microscopic features that can suggest phaeohyphomycosis: branched (gray dart) pigmented filaments with visible septate (black arrow). The specific morphological features of Rhinocladiella mackenziei have been well described elsewhere [2,8,9]. HES, hematoxylin–eosin saffron; MRI, magnetic resonance imaging. (TIF) [file pntd.0009563.s001.tif]
