# Supplementary material for: Morocco as a possible source for acquisition of Rhinocladiella mackenziei
Source: PLoS Negl Trop Dis. 2021 Aug 19;15(8):e0009563. doi: 10.1371/journal.pntd.0009563 (PMC8376069; doi:10.1371/journal.pntd.0009563)
Supplement: S1 Table — To identify cases, we searched in PubMed using the following keywords: “Rhinocladiella mackenzieie,” “Ramichloridium obovoideum,” “Ramichloridium mackenziei,” and “Ramichloridium obovoidea.” We searched from database inception onward without language restrictions. The last search was conducted on May 15, 2020. (DOCX) [file pntd.0009563.s002.docx]

**S1 Table. Cases reported in Fig 1.**

| Case | Reference | Countries at risk |
| --- | --- | --- |
| 1 | [1] | Saudi Arabia |
| 2 | [1] | Saudi Arabia |
| 3 | [1] | Saudi Arabia |
| 4 | [2] | Saudi Arabia |
| 5 | [2] | Saudi Arabia |
| 6 | [2] | Israel |
| 7 | [2] | Qatar (past travel to India) |
| 8 | [2] | Oman |
| 9 | [2] | United Arab Emirates |
| 10 | [2] | Saudi Arabia |
| 11 | [3] | Saudi Arabia |
| 12 | [4] | Kuwait |
| 13 | [5] | Saudi Arabia |
| 14 | [5] | Saudi Arabia |
| 15 | [6] | Saudi Arabia |
| 16 | [6] | Saudi Arabia |
| 17 | [7] | Kuwait (Egyptian origin) |
| 18 | [8] | Saudi Arabia |
| 19 | [9] | Saudi Arabia |
| 20 | [10] | India |
| 21 | [11] | Qatar |
| 22 | [12] | Afghanistan (living in France) |
| 23 | [13] | Saudi Arabia |
| 24 | [14] | Pakistan |
| 25 | [14] | Pakistan |
| 26 | [14] | Pakistan |
| 27 | [14] | Pakistan |
| 28 | [14] | Pakistan |
| 29 | [14] | Pakistan |
| 30 | [15] | Somalia (living in Denmark) |
| 31 | [16] | Iran |
| 32 | [17] | India (living in the UK; past travels to Kenya, Egypt and Turkey) |
| 33 | [18] | Iran |
| 34 | [19] | United Arab Emirates (living in the UK, past travels to Irak and Jordan) |
| 35 | Current study | Morocco (living in France) |
| 36 | Current study | Morocco (living in France) |

[1] Naim-ur-Rahman, Mahgoub ESh, Chagla AH. Fatal brain abscesses caused by Ramichloridium obovoideum: Report of three cases. Acta Neurochir (Wien) 1988;93:92–5. https://doi.org/10.1007/BF01402887.

[2] Campbell CK, Al-Hedaithy SSA. Phaeohyphomycosis of the brain caused by *Ramichloridium mackenziei* sp. nov. in Middle Eastern Countries. Med Mycol 1993;31:325–32. https://doi.org/10.1080/02681219380000391.

[3] Sutton DA, Slifkin M, Yakulis R, Rinaldi MG. U.S. Case Report of Cerebral Phaeohyphomycosis Caused by Ramichloridium obovoideum (R. mackenziei): Criteria for Identiﬁcation, Therapy, and Review of Other Known Dematiaceous Neurotropic Taxa. J CLIN MICROBIOL 1998;36:8.

[4] Podnos YD, Anastasio P, Maza LDL, Kim RB. Cerebral Phaeohyphomycosis Caused by Ramichloridium obovoideum (Ramichloridium mackenziei). Neurosurgery 1999;45:372–4. https://doi.org/10.1097/00006123-199908000-00034.

[5] Kashgari TQ, Al-Miniawi H, Moawad Hanna MK. Cerebral Phaeohyphomycosis Caused by *Ramichloridium Mackenziei* in the Eastern Province of Saudi Arabia. Ann Saudi Med 2000;20:457–60. https://doi.org/10.5144/0256-4947.2000.457.

[6] Kanj SS, Amr SS. Ramichloridium mackenziei brain abscess: report of two cases and review of the literature. Med Mycol 2001:6.

[7] Khan ZU, Lamdhade SJ. Additional case of Ramichloridium mackenziei cerebral phaeohyphomycosis from the Middle East. Med Mycol 2002:5.

[8] Al-abdely HM, Alkhunaizi AM, Al-tawfiq JA, Hassounah M, Rinaldi MG, Sutton DA. Successful therapy of cerebral phaeohyphomycosis due to *Ramichloridium mackenziei* with the new triazole posaconazole. Med Mycol 2005;43:91–5. https://doi.org/10.1080/13693780400011104.

[9] Amr SS, Al-Tawfiq JA. Aspiration cytology of brain abscess from a fatal case of cerebral phaeohyphomycosis due toRamichloridium mackenziei. Diagn Cytopathol 2007;35:695–9. https://doi.org/10.1002/dc.20724.

[10] Badali H, Chander J, Bansal S, Aher A, Borkar SS, Meis JF, et al. First Autochthonous Case of Rhinocladiella mackenziei Cerebral Abscess Outside the Middle East. J Clin Microbiol 2010;48:646–9. https://doi.org/10.1128/JCM.01855-09.

[11] Taj-Aldeen SJ, Almaslamani M, Alkhalf A, Al Bozom I, Romanelli AM, Wickes BL, et al. Cerebral phaeohyphomycosis due to *Rhinocladiella mackenziei* (formerly *Ramichloridium mackenziei* ): a taxonomic update and review of the literature. Med Mycol 2010;48:546–56. https://doi.org/10.3109/13693780903383914.

[12] Cristini A, Garcia-Hermoso D, Celard M, Albrand G, Lortholary O. Cerebral Phaeohyphomycosis Caused by Rhinocladiella mackenziei in a Woman Native to Afghanistan. J Clin Microbiol 2010;48:3451–4. https://doi.org/10.1128/JCM.00924-10.

[13] Al-Tawfiq JA, Boukhamseen A. Cerebral phaeohyphomycosis due to Rhinocladiella mackenziei (formerly Ramichloridium mackenziei): Case presentation and literature review. J Infect Public Health 2011;4:96–102. https://doi.org/10.1016/j.jiph.2011.01.001.

[14] Jabeen K, Farooqi J, Zafar A, Jamil B, Faisal Mahmood S, Ali F, et al. Rhinocladiella mackenziei as an Emerging Cause of Cerebral Phaeohyphomycosis in Pakistan: A Case Series. Clin Infect Dis 2011;52:213–7. https://doi.org/10.1093/cid/ciq114.

[15] Pedersen MB, Zhao Y, Arendrup MC, Bendix K, Bojsen-Møller M, Mølle I, et al. Co-existence of cerebral infection with Rhinocladiella mackenziei and primary central nervous system lymphoma in a HIV-negative patient: LETTER TO THE EDITOR. APMIS 2011;119:221–3. https://doi.org/10.1111/j.1600-0463.2010.02713.x.

[16] Didehdar M, Gokanian A, Sofian M, Mohammadi S, Mohammadi R, Aslani N, et al. First fatal cerebral phaeohyphomycosis due to Rhinocladiella mackenziei in Iran, based on ITS rDNA. J Mycol Médicale 2015;25:81–6. https://doi.org/10.1016/j.mycmed.2014.11.003.

[17] Yusupov N, Merve A, Warrell CE, Johnson E, Curtis C, Samandouras G. Multiple brain abscesses caused by Rhinocladiella mackenziei in an immunocompetent patient: a case report and literature review. Acta Neurochir (Wien) 2017;159:1757–63. https://doi.org/10.1007/s00701-017-3141-0.

[18] Mohammadi R, Mohammadi A, Ashtari F, Khorvash F, Hakamifard A, Vaezi A, et al. Cerebral phaeohyphomycosis due to *Rhinocladiella mackenziei* in Persian Gulf region: A case and review. Mycoses 2018;61:261–5. https://doi.org/10.1111/myc.12734.

[19] Hardman N, Young N, Hobson R, Sandoe J, Wellberry‐Smith M, Thomson S, et al. Prolonged survival after disseminated *Rhinocladiella* infection treated with surgical excision and posaconazole. Transpl Infect Dis 2020;22. https://doi.org/10.1111/tid.13264.
